# Supplementary figures and images for: High resistance to climatic variability in a dominant tundra shrub species
Source: PeerJ. 2019 Jun 5;7:e6967. doi: 10.7717/peerj.6967 (PMC6556101; doi:10.7717/peerj.6967)

## Oceanic to Continental climate

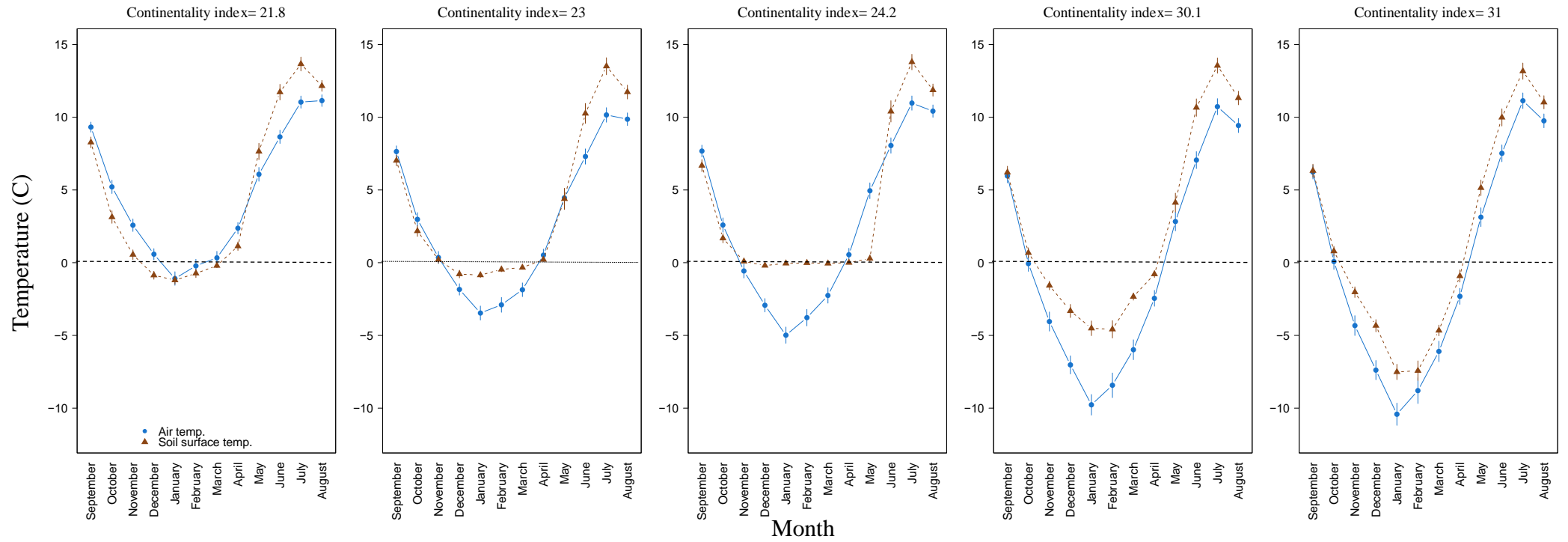

Supplement: Figure S1 — Mean air temperature extracted from publicly available online databases(i.e., http://www.senorge.no) and soil surface temperature extracted from on-site temperature loggers, along the continentality gradient and across all six study years (2011–2016). [file peerj-07-6967-s002.pdf]

a) NMDS Plot for study years

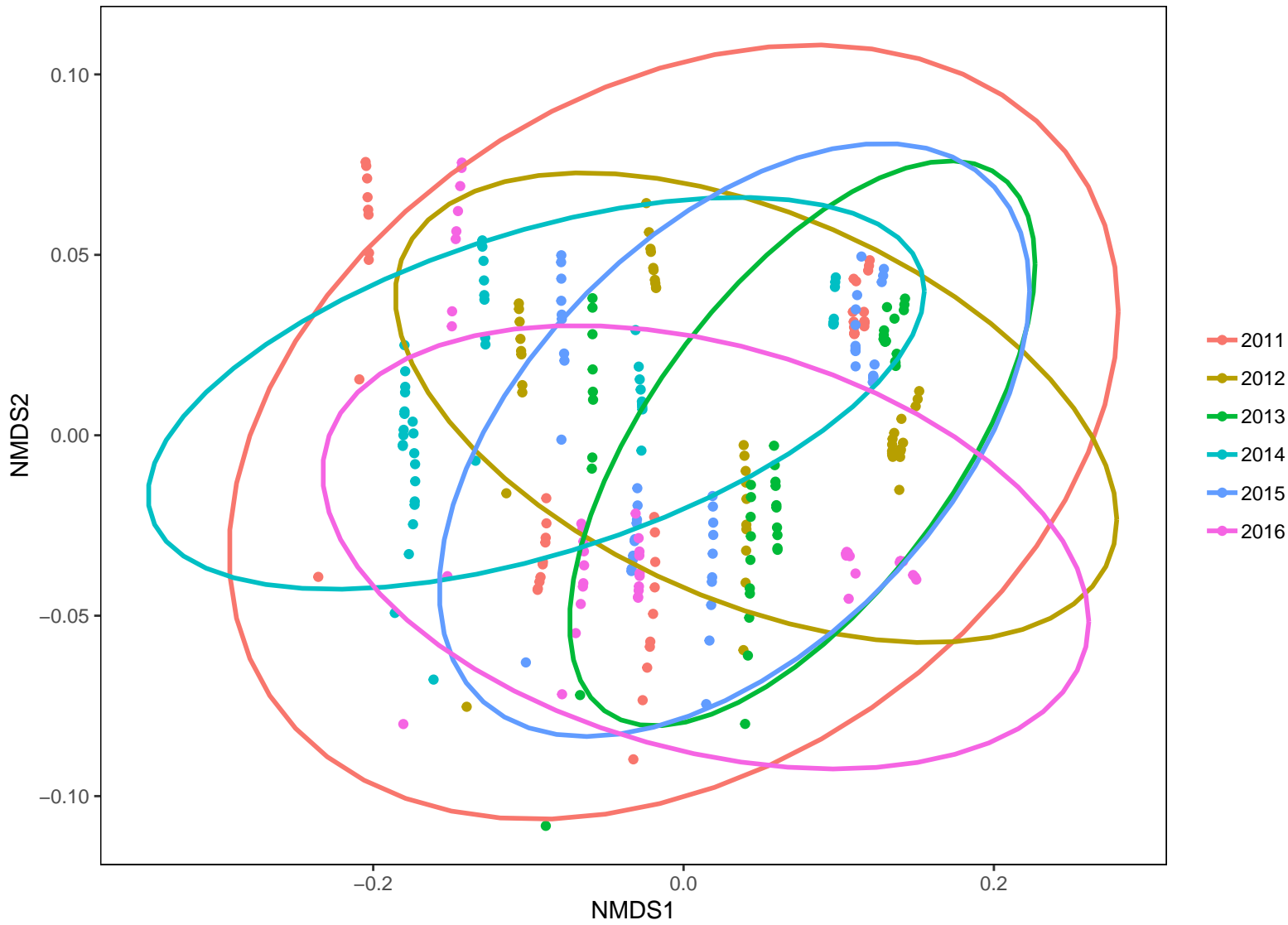

b) NMDS Plot for Continental Index

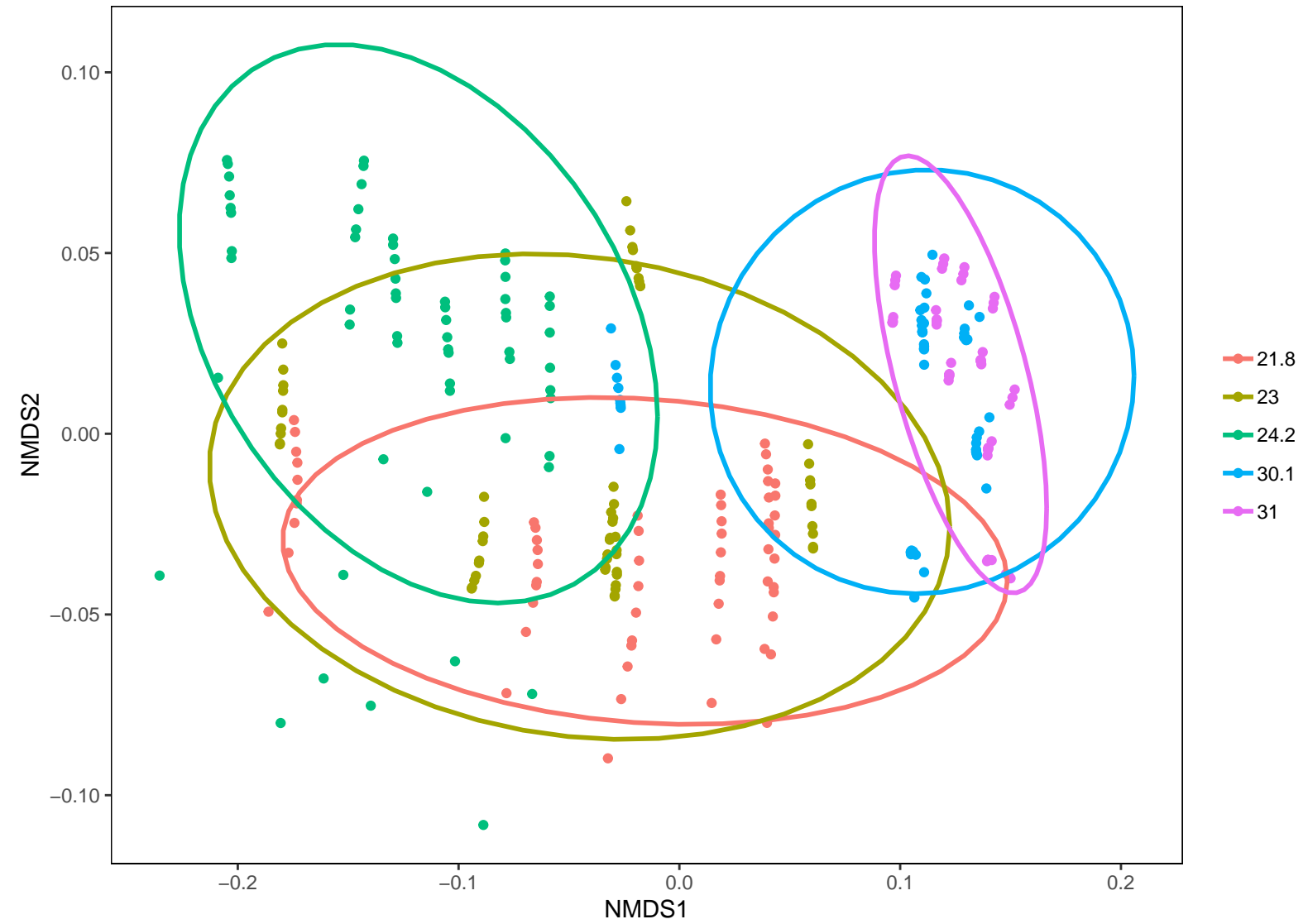

Supplement: Figure S2 — (A) through-out the study years (i.e. 2011–2016), and (B) the sites along the gradient from oceanic to continental climate (i.e., 21.8-31) [file peerj-07-6967-s003.pdf]
