# Supplementary material for: High resistance to climatic variability in a dominant tundra shrub species
Source: PeerJ. 2019 Jun 5;7:e6967. doi: 10.7717/peerj.6967 (PMC6556101; doi:10.7717/peerj.6967)
Supplement: Table S1 — Significance values =∗p < 0.05, ∗∗p < 0.01 [file peerj-07-6967-s004.docx]

Supplemental table S1. Pearson correlation values between the predictor variables. Significance values= *p<0.05,**p<0.01

|  | FD | ST | GDD+1 | GDD+2 | GDD+5 | GSL | GSP | NGSP |
| --- | --- | --- | --- | --- | --- | --- | --- | --- |
| Biomass | 0.30* | -0.03 | -0.07 | -0.06 | -0.05 | -0.05 | 0.027 | -0.27* |
| FD |  | -0.11* | 0.05 | 0.04 | 0.03 | 0.24** | 0.27* | -0.84** |
| ST |  |  | 0.81** | 0.82** | 0.83** | 0.09 | -0.11* | 0.12* |
| GDD+1 |  |  |  |  |  | 0.67** | 0.17** | -0.05 |
| GDD+2 |  |  |  |  |  | 0.66** | 0.15** | -0.04 |
| GDD+5 |  |  |  |  |  | 0.68** | 0.13** | -0.03 |
| GSL |  |  |  |  |  |  | 0.46** | -0.34** |
| GSP |  |  |  |  |  |  |  | -0.37** |
