# Supplementary material for: High resistance to climatic variability in a dominant tundra shrub species
Source: PeerJ. 2019 Jun 5;7:e6967. doi: 10.7717/peerj.6967 (PMC6556101; doi:10.7717/peerj.6967)
Supplement: Table S2 — Response variables were mean shoot length and mean berry frequency while predictor variables were mean number of freezing days (FD), growing degree days (GDD+1) and their interaction, from five sites over six years [file peerj-07-6967-s005.docx]

Supplemental table S2. ANOVA table showing results from the mixed effects models. Response variables were mean shoot length and mean berry frequency while predictor variables were mean number of freezing days (FD), growing degree days (GDD+1) and their interaction, from five sites over six years.

|  | Mean shoot length | | | Mean berry frequency | | |
| --- | --- | --- | --- | --- | --- | --- |
|  | DF | F-value | p-value | DF | F-value | p-value |
| FD | 1,237 | 27.79 | **<0.01** | 1,237 | 6.90 | **<0.01** |
| GDD | 1,237 | 5.67 | **0.018** | 1,237 | 22.96 | **<0.01** |
| FDxGDD | 1,237 | 20.79 | **<0.01** | 1,237 | 40.63 | **<0.01** |
